# Propionic acid counteracts the inflammation of human subcutaneous adipose tissue: a new avenue for drug development

**DOI:** 10.1007/s40199-019-00294-z

**Published:** 2019-09-11

**Authors:** Sa’ad Al-Lahham, Farhad Rezaee

**Affiliations:** 1 Department of Biomedical Sciences, Faculty of Medicine and Health Sciences, An-Najah National University, Nablus, Palestine; 2 Department of Gastroenterology and Hepatology, Erasmus Medical Center, University of Rotterdam, Rotterdam, The Netherlands

**Keywords:** Propionic acid, Subcutaneous adipose tissue, Inflammation, Gi/o proteins coupled receptors, IP-10 and TNF-alpha, macrophage

## Abstract

Adipose tissue is a primary site of obesity-induced inflammation, which has been emerging as an important contributor to obesity associated disorders. The factors influencing adipose tissue-induced inflammation and the resulting pathophysiological events remain poorly understood. However, dietary fiber consumptions appear to be protective. Short-chain fatty acids such as propionic acid (PA) are the principal products of the dietary fiber fermentation by microbiota. Therefore, we aim to investigate the influence of PA on inflammation, lipogenesis and glucose uptake markers from human subcutaneous adipose tissue (SAT). We showed that the treatment of SAT with PA resulted in a significant downregulation of inflammatory parameters (e.g. TNF-α and IP-10) and macrophage markers (e.g. CD163 and MMP-9). The expression levels of PA receptors (i.e. G protein coupled receptor-41 and -43) in human primary adipocytes were very low in comparison with SAT and macrophages. Upon PA treatment, no anti-inflammatory effect was observed in human adipocytes. PA significantly upregulated the expression of lipoprotein lipase (LPL), sterol regulatory-element-binding protein-1c (SREBP-1c) and glucose transporter 4 (GLUT-4), which are associated with lipogenesis and glucose uptake. We also showed that the observed anti-inflammatory effects of PA on SAT were partly mediated by Gi/o protein coupled receptor. Our data suggests that PA anti-inflammatory effects on SAT are mediated partly via Gi/o proteins, leading to the improved expression of factors associated with lipogenesis and glucose uptake. These responses appeared to be not mediated by adipocytes; but most probably by macrophages. The current study provides new knowledge, which can be used as a potential new avenue for drug development in preventing obesity-related inflammation and metabolic disorders in future.

**Graphical abstract:**
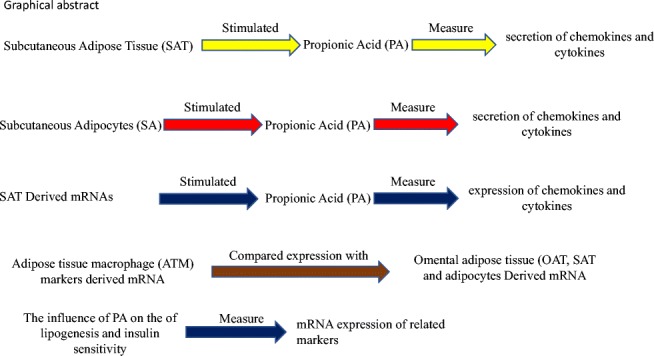
Schematic presentation of study flow and the components of the investigation. In this study the effect of propionic acid (PA) on inflammation investigated in human subcutaneous adipose tissue (SAT), human primary adipocytes and the expression of a few hallmark inflammatory components produced by SAT and human adipocytes.

Schematic presentation of study flow and the components of the investigation. In this study the effect of propionic acid (PA) on inflammation investigated in human subcutaneous adipose tissue (SAT), human primary adipocytes and the expression of a few hallmark inflammatory components produced by SAT and human adipocytes.

## Introduction

Obesity has reached epidemic proportions and is still escalating at an alarming rate worldwide. World Health Organization reported that population living on earth is approximately 7.77.7 billion of which more than 1.9 billion adults (~39%) with overweight and over 600 million with obese (~13%) [1]. In Palestine the prevalence of obesity has been shown to be approximately 4 times among women (49%) and 2 times among men (30%) higher than the worldwide observed prevalence [2]. 

Obesity is associated with chronic activation of low-grade inflammation [3], which is implicated in the pathogenesis of obesity-associated diseases including insulin resistance, type-2 diabetes (T2D) [4, 5] and cardiovascular disease [6, 7]. The etiology of obesity and low-grade inflammation is complex and involves intrinsic and extrinsic factors. Recently, it has been shown that specific members of microbiota in humans, in particular *Firmicutes*, are associated with obesity and its associated afflictions [8–11]. 

Furthermore, the colonization of germ-free mice with microbiota derived from obese mice results in significantly greater adiposity than colonization with microbiota from lean mice [12]. Conversely, prebiotic diets such as fructans [13] are associated with general better health, including the decrease in body weight, fat mass and the severity of T2D [14–16]. The factors that influence the composition and metabolism of intestinal microbiota and obesity and its related inflammation and pathophysiology remain, however, obscure at best.

Fermentation of dietary fiber/ resistant starch by colonic microbiota is a primary source for the production of short-chain fatty acids (SCFAs), in particular acetic, butyric and propionic acids. It has been demonstrated that SCFAs influence the physiology of humans such as inhibition of inflammation, protection from cancer and promotion of satiety [17–20]. Recently it has been shown that mice deficient in G protein–coupled receptor 43 (GPR43) have exacerbated and poorly resolving inflammation in animal models of arthritis, allergic airway inflammation and colitis [21]. Germ-free mice model of arthritis has also showed increased inflammation and a much slower resolution of inflammation when compared to conventionally raised mice. GPCR43 is a receptor of SCFAs and consequently providing acetate in the drinking water has reduced inflammation in these mice. Therefore, one could envisage that SCFAs, including PA, may constitute the elusive link between the host and microbiota.

Obesity triggers inflammation in Adipose tissue (AT), which in turn implicated in pathophysiological events such as T2D. AT is also primary organ involved inobesity [20, 22–25]. In the current study, we determined the anti-inflammatory effect of PA on SAT and adipocytes [26].

## Methods

### Materials

Gentamycin, glucose and PA were purchased from Sigma. M199 media was purchased from Invitrogen. Preadipocytes and their media were purchased from PromoCell. Cluster of differentiation 16A (CD16A), Cluster of differentiation 31 (CD31), matrix metalloproteinase 9 (MMP-9), GPCR41 and GPCR43 primers were purchased from Applied Biosystems.

### Human adipose tissue (AT) and cell culture

AT explants were obtained from human subjects who underwent surgery for disorders. None of the human subjects had diabetes and the age average was 48, and Body Mass Index average was 28 kg/m^2^. This study was approved by the An-Najah National University, Institutional Review Board (IRB) (approves, monitors, and research involving humans) Committee and informed written consent was obtained from all subjects. AT culture was performed as described previously [26, 27] with slight modifications. After the last washing step, tissue explants were incubated for 24 h with or without 3 mM PA. AT explants were pre-incubated with pertussis toxin (PTX) (100 ng/mL) for 2 h. Subsequently, AT explants were treated with PA (3 mM) for 24 h. All tissues were snap frozen in liquid nitrogen and stored at −80 °C until RNA isolation. Secretome (Media fraction collected from cultured tissues) was stored at −80 °C meant for ELISA and multi ELISA measurements. With respect to human preadipocytes, they were cultured and differentiated into adipocytes according to PromoCell instructions.

### RT-PCR analysis

Total RNA was isolated by the RNeasy lipid tissue mini kit and cDNA was synthesized using the Quantitect kit (Qiagen). Relative quantification of genes was performed in triplicates as described earlier [26, 27]. Briefly, the primers’ pairs and probes used in this study are displayed in Table 1. Stability of several housekeeping genes was assessed by geNorm analysis software (http://medgen.ugent.be/~jvdesomp/genorm/) [28]. GAPDH was chosen as the most stable housekeeping gene expressed in AT.

**Table 1:** The sequences of the primers

Primer ID	Primer sequence (5′➔3′)
GAPDH forward	GGT GAA GGT CGG AGT CAA CG
GAPDH reverse	ACC ATG TAG TTG AGG TCA ATG AAG G
GAPDH probe	CGC CTG GTC ACC AGG GCT GC
GLUT4 forward	GCT GTG GCT GGT TTC TCC AA
GLUT4 reverse	CCC ATA GCC TCC GCA ACA TA
GLUT4 probe	CGA GCA ACT TCA TCA TTG GCA TGG GTT
LPL forward	TGG AGA TGT GGA CCA GCT AGT G
LPL reverse	CAG AGA GTC GAT GAA GAG ATG AAT G
LPL probe	CTC CCA CGA GCG CT
SREBP1c forward	GGA TTG CAC TTT CGA AGA CAT G
SREBP1c reverse	AGC ATA GGG TGG GTC AAA TAG G
SREBP1c probe	CAG CTT ATC AAC AAC CAA GAC AGT GAC TTC CC
CD163 forward	TGC AGA AAA CCC CAC AAA AAG
CD163 reverse	CAA GGA TCC CGA CTG CAA TAA
CD163 probe	AAC AGG TCG CTC ATG CCG TCA GTC A
CD16	Hs01569121_m1*
CD31	Hs01065282_m1*
MMP-9	Hs00234579_m1*
GPCR41	Hs00271131_s1*
GPCR43	Hs00271142_s1*

*, ID numbers of primer sets from Applied Biosystems

#### The analysis of chemokines and cytokines proteins in secreted fraction

Secreted chemokines and cytokines were measured in culture media by multiplex-ELISA assay according to the manufacturer’s instructions (Bio-Rad).

### Statistics

Comparison between two groups was performed by two-sided paired Student’s t test, while the rest were analyzed via either one- or two-ways ANOVA. Results were considered to be statistically significant at *P <* 0.05.

## Results

### Propionic Acid (PA) effect on inflammation in human subcutaneous adipose tissue (SAT)

**Fig. 1:**
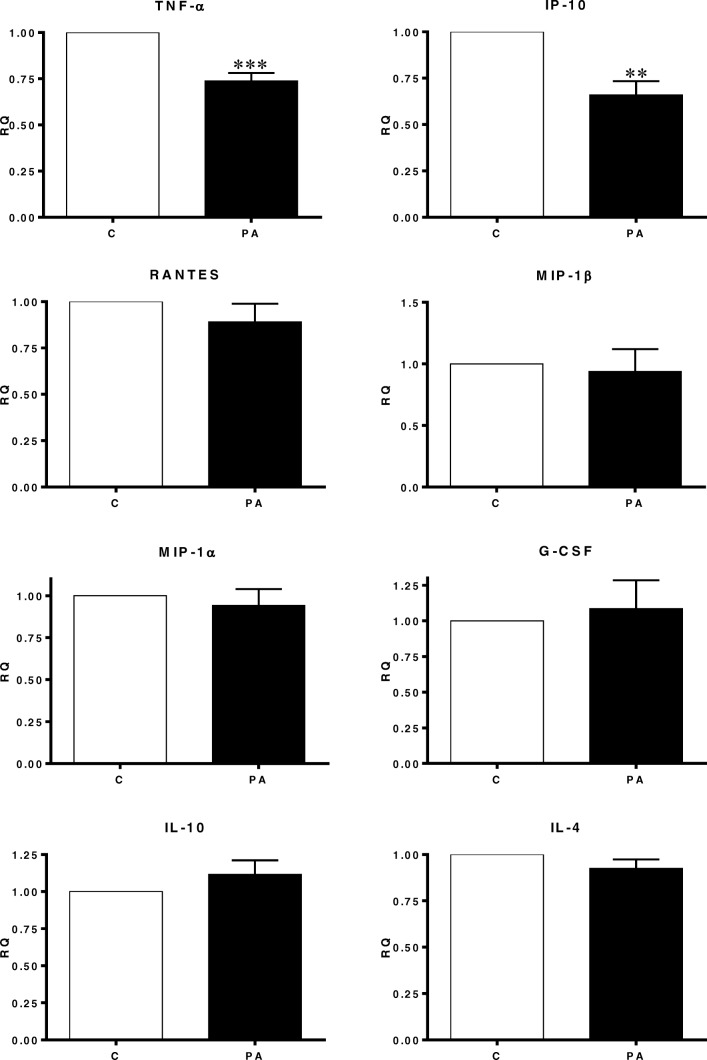
The influence of Propionic Acid (PA) on the secretion of chemokines and cytokines by subcutaneous adipose tissue (SAT). SAT explants of each subject were incubated in triplicate with or without 3 mM PA for 24 h. Secreted quantities of chemokines and cytokine in the media were determined by multiplex-ELISA. Results are expressed as relative quantities (RQ) and compared to the control (without PA; C). (*N* = 10). **, *P* < 0.01, ***, *P* < 0.001 vs. control (without PA; C). M, mean

**Fig. 2:**
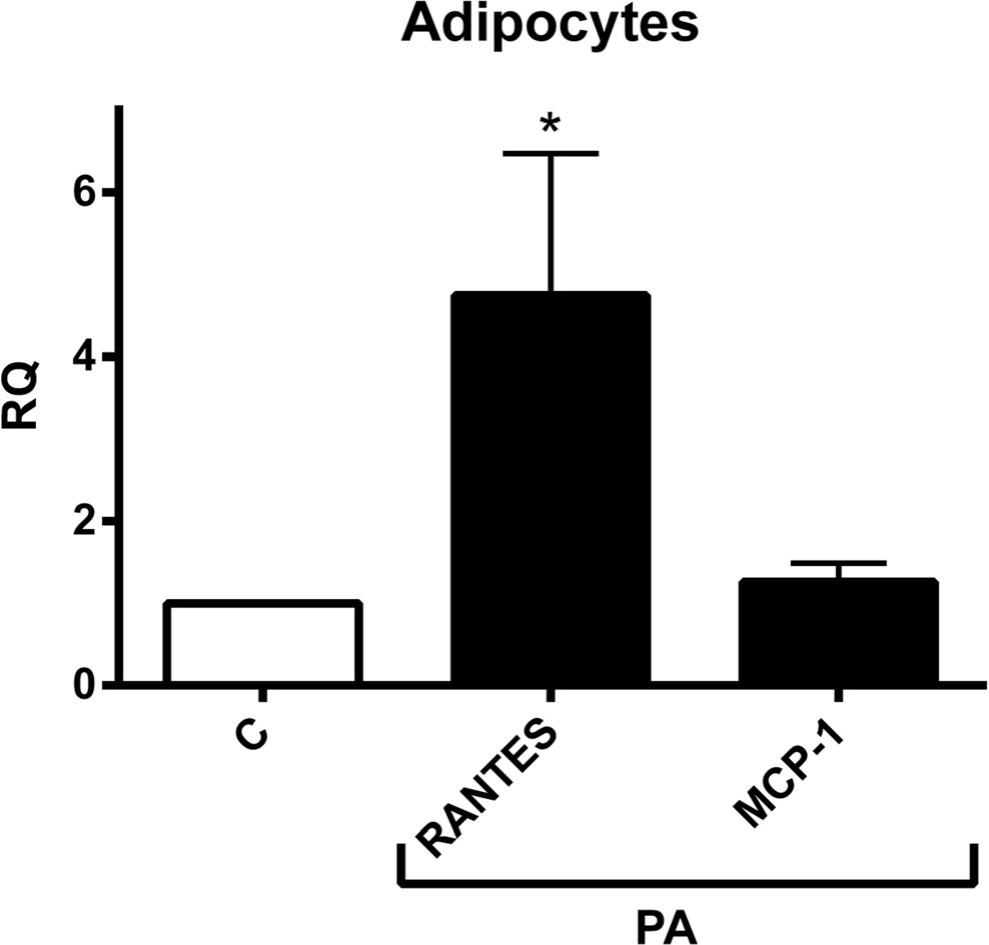
The effect of Propionic Acid (PA) on the secretion of chemokines and cytokines by human adipocytes. Adipocytes incubated in triplicate with or without 3 mM PA for 24 h. Secreted quantities of chemokines and cytokine in the media were determined by multiplex-ELISA. Only RANTES and MCP-1 were detected and the rest weren’t. Results are expressed as relative quantities (RQ) compared to the control (without PA; C). *, *P* < 0.05 vs. control (without PA; C). M, mean

Human SAT explants were derived from 10 women. The averages of their BMI, WHR, WC and age were approximately 28, 0.84, 86 and 48 respectively. As demonstrated in Fig. 1, PA treatment significantly downregulated the secretion of tumor necrosis factor-α (TNF-α) and interferon-gamma-induced protein (IP-10) by approximately 30%. On the contrary, the expression of Macrophage Inflammatory Proteins-1α and -1β (MIP-1α and MIP-1β), Regulated on Activation Normal T Cell Expressed and Secreted (RANTES), interleukins (IL-1β, IL-4, IL-10) were remained unchanged upon the PA stimulation. Il-12 and 13 were not detected in human SAT. In adipocytes the secretion of TNF-α, IP-10, MIP-1α, MIP-1β, IL-1β, IL-4, IL-10, IL-12 and IL-13 was not detected and monocyte chemoattractant protein-1 (MCP-1) was not affected. Whereas and unexpectedly, RANTES was increased approximately by 3 folds after PA treatment (Fig. 2).

## Propionic Acid (PA) inhibited the expression of adipose tissue macrophage (ATM) markers

**Fig. 3:**
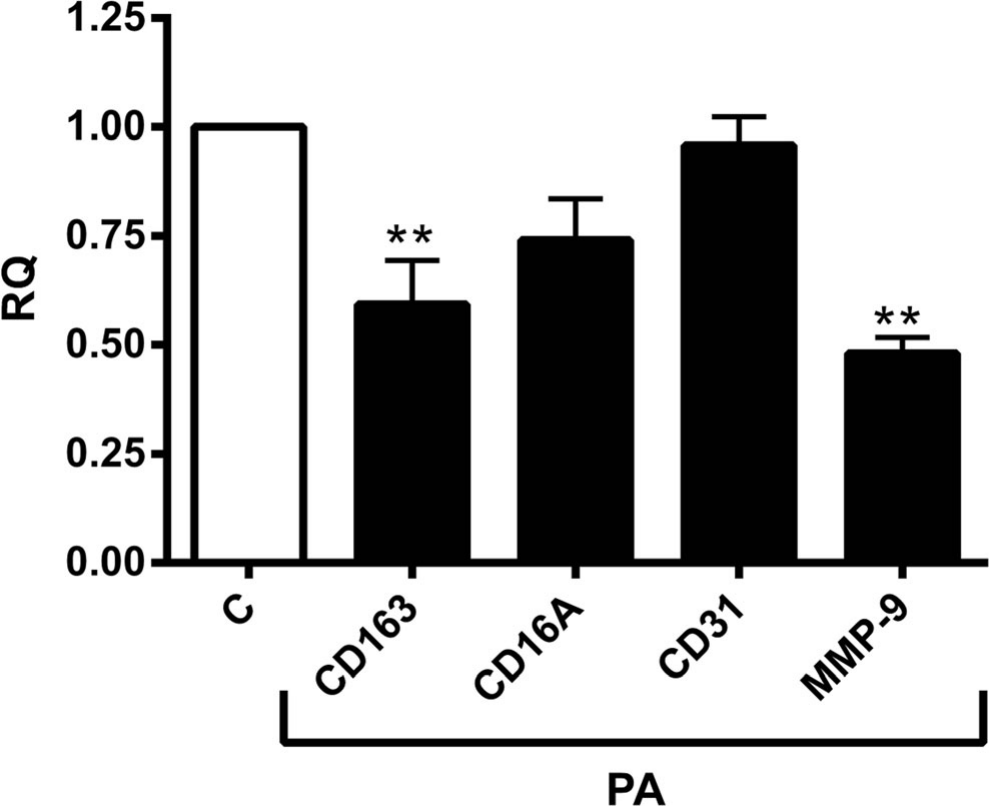
The effect of propionic Acid (PA) on the mRNA expression of adipose tissue macrophage (ATM) related markers. Human subcutaneous adipose tissue (SAT) explants of each subject were incubated in triplicate with or without 3 mM PA for 24 h. PA downregulated all of the ATM markers, i.e. CD16, CD163 and MMP-9. The mRNA expression levels were determined by RT-PCR and expressed as relative quantities (RQ) and compared to the control (without PA; C). (*N* = 10) *, *P* < 0.05 vs. control (without PA; C). M, mean

PA inhibited mRNA expression of ATM markers; CD163, MMP-9 and CD16A in SAT by approximately 40%, 52% and 25% respectively, while it had no effect on CD31 mRNA expression as depicted in Fig. 3.

## The comparison of ATM markers expression levels between omental adipose tissue (OAT), subcutaneous adipose tissue (SAT) and adipocytes

**Fig. 4:**
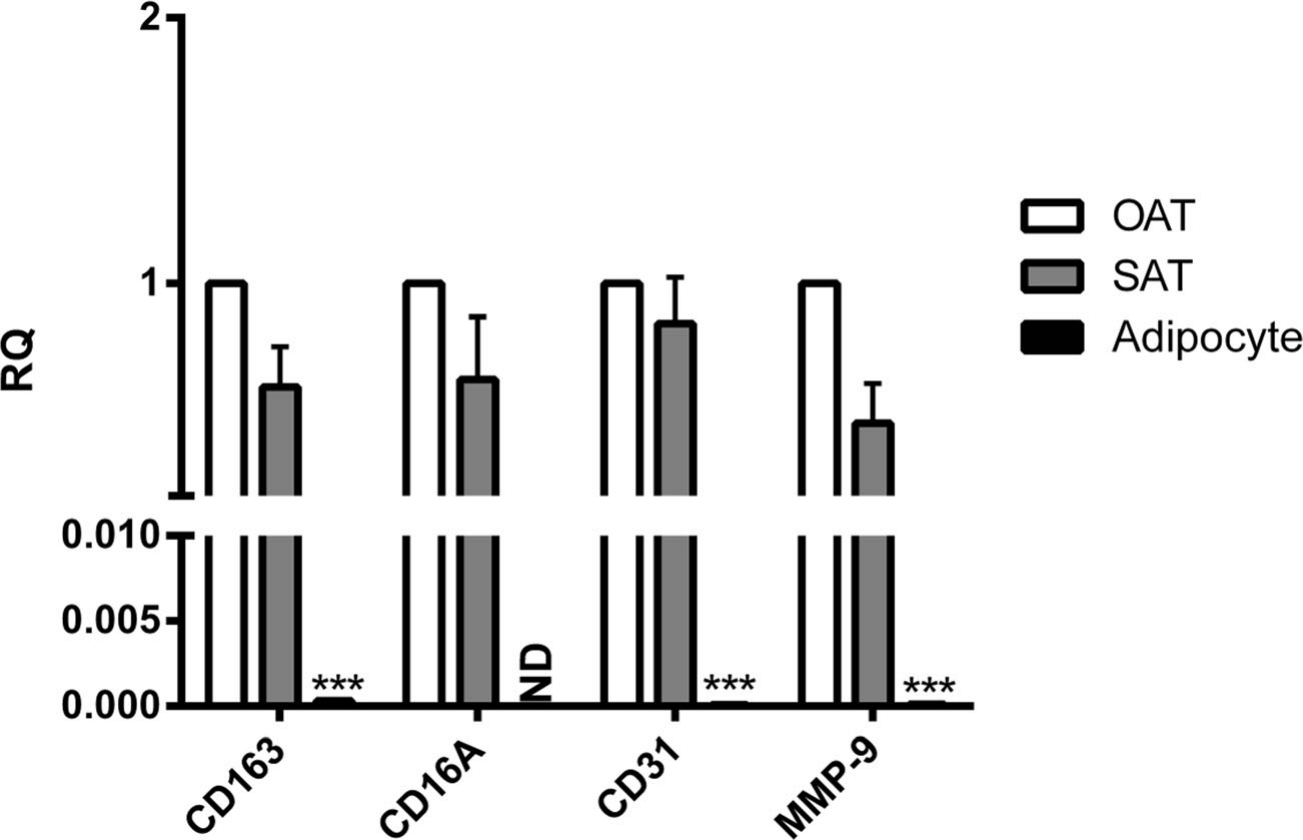
Comparing mRNA expression of adipose tissue macrophage (AMT) markers produced in omental adipose tissue (OAT), SAT and adipocytes**.** mRNA was isolated from untreated explants and adipocytes in triplicate. mRNA expression levels were determined by RT-PCR and expressed as relative quantities (RQ) compared to the control (OAT). (N = 10). *, *P* < 0.05, ***, *P* < 0.001 vs. OAT. M, mean

mRNA levels of CD163, CD31 and MMP-9 in OAT were significantly 3201-, 9857- and 8553-folds higher than in human adipocytes, respectively. Whereas CD16 was not detected in adipocytes. As compared to SAT, MMP-9 mRNA levels were found to be approximately 50% higher in OAT; But there were no significant differences between OAT and SAT with respect to CD163, CD16 and CD31 mRNA levels (Fig. 4).

## Comparison of GPCR 41 and 43 expression levels between SAT, adipocytes and macrophages

**Fig. 5:**
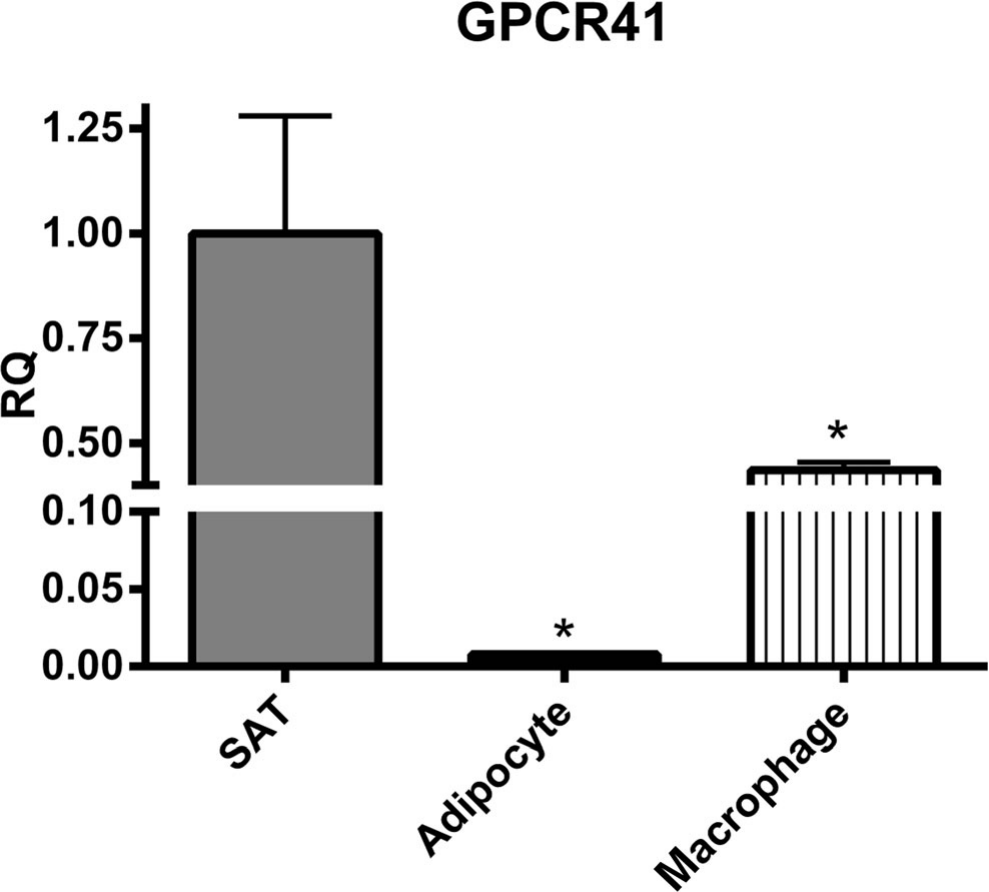
Comparing mRNA levels of GPCR41 and 43 produced in human subcutaneous adipose tissue (SAT), adipocytes and macrophages**.** mRNA was isolated from untreated explants and adipocytes in triplicate. mRNA expression levels were determined by RT-PCR and shown as relative quantities (RQ) compared to the control (SAT). (N = 10). *, *P* < 0.05 vs. SAT. M, mean

As shown in Fig. 5, mRNA level of PA receptor GPCR41 was significantly higher in SAT than in adipocytes and macrophages by 125- and 4-folds respectively. The quantity of GPCR43 in SAT was similar to the quantity expressed in macrophages; however, it was tremendously lower in adipocytes (~1050-folds).

## Propionic acid positively affects key metabolic genes in human adipose tissue explants

**Fig. 6:**
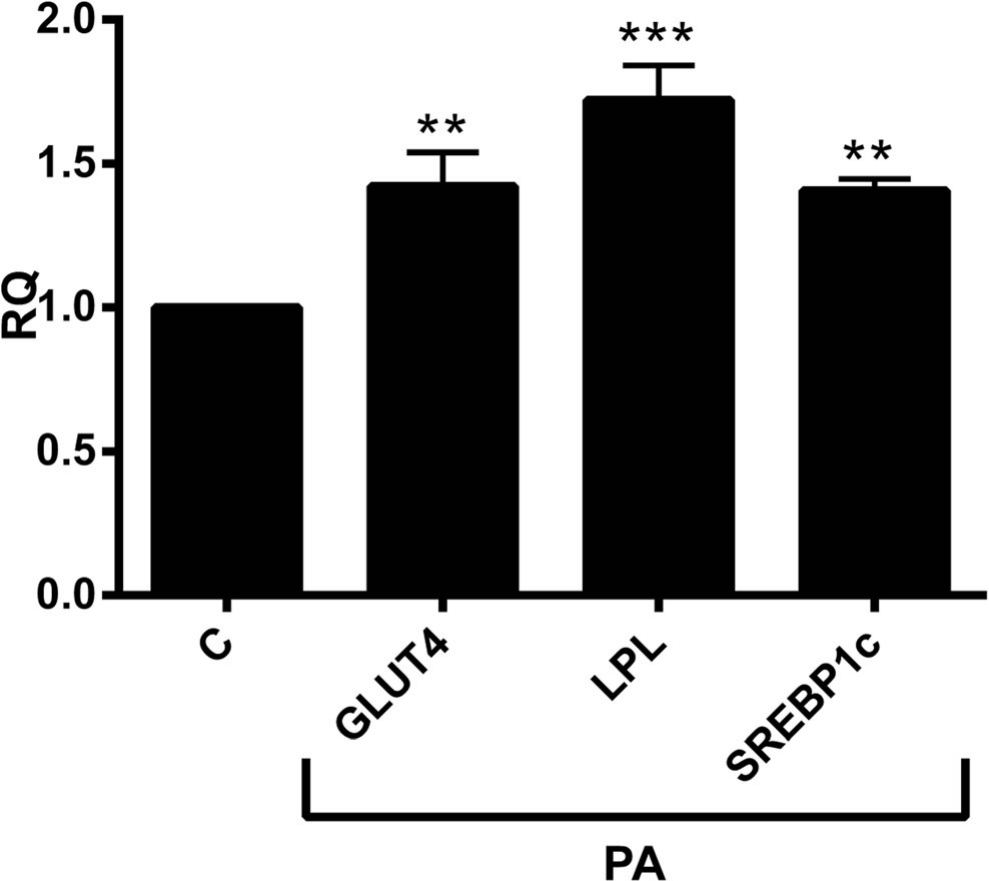
The role of Propionic Acid (PA) on the mRNA expression of lipogenesis and insulin sensitivity related markers. Human subcutaneous adipose tissue (SAT) explants of each subject were incubated in triplicate with or without 3 mM PA for 24 h. PA upregulated the expression of lipoprotein lipase (LPL), SREBP-1c and GLUT-4. The mRNA expression levels were determined by RT-PCR and shown as relative quantities (RQ) compared to the control (without PA; C). (N = 10). *, *P* < 0.05, **, *P* < 0.01, ***, *P* < 0.001 vs. control (without PA; C). M, mean

As we observed above, PA had anti-inflammatory effect on the AT. To investigate whether anti-inflammatory effect of PA negatively influences the associated-markers of metabolic syndrome, such as lipogenesis and insulin sensitivity, we incubated SAT explants with or without PA in triplicates. As shown in Fig. 6, the lipoprotein lipase (LPL), sterol regulatory-element-binding protein-1c (SREBP-1c) and glucose transporter 4 (GLUT-4) mRNA levels were highly upregulated in all explants upon PA treatment by approximately 72%, 41% and 42% respectively (Fig. 6).

## Gi/o-protein coupled receptors role in the anti-inflammatory effect of PA

**Fig. 7:**
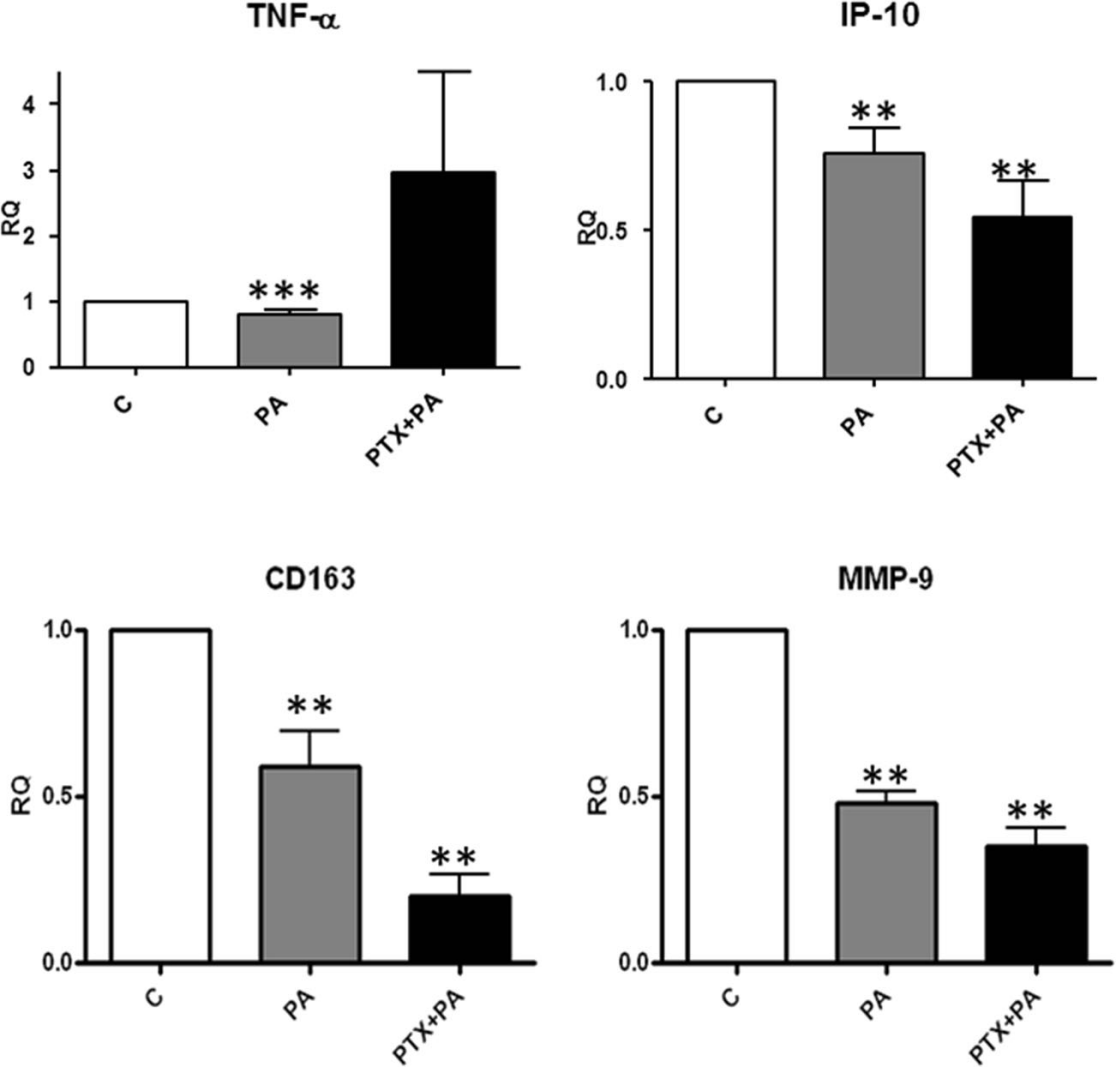
Involvement of G protein coupled receptor(s). The involvement of Gi/o coupled receptors in mediating Propionic Acid (PA) effects on adipokines expression was determined by blocking the Gi /o signaling pathway with pertussis toxin (PTX). Human subcutaneous adipose tissue (SAT) explants of each subject were incubated in triplicate with PTX (100 ng / ml) for 2 h before incubation for 24 h with or without 3 mM PA. Protein (TNF-α and IP-10) and mRNA expression (CD163 and MMP-9) levels were determined using ELISA and RT-PCR respectively. Results were depicted as relative quantities compared with controls (without PA). (*N* = 5). **, *P* < 0.01, ***, *P* < 0.001 vs. control (without PA; C). M, mean

PA is a ligand of both GPCR41 and GPCR43, which both activate Gi/o-proteins to prime PA signaling [26–28]. To determine the role of Gi/o pathway in the response of inflammatory markers to PA treatment in SAT the SAT biopsies were pretreated with PTX for 2 h to block the Gi/o pathway and they were subsequently treated with or without 3 mM PA for 24 h. As displayed in Fig. 7, the inhibition of TNF-α release by PA was completely abolished by PTX pretreatment, while the PA-induced reduction in IP-10, CD163 and MMP-9 expression was not affected by PTX.

## Discussion

SCFAs are mainly metabolites of gut microbiota fermentation of escaped undigested food. A numerous of studies has been shown that SCFAs inhibit inflammation with focus on butyrate and to a lesser extent on acetate and PA, [16]. In our previous study we have shown that PA has anti-inflammatory properties in OAT [26, 27]. The anti-inflammatory properties of PA were confirmed by showing that the mice remain lean even when put on a high-fat diet in mice overexpressing PA receptor (GPR43) specifically in OAT. Moreover, these mice exhibit a decrease of macrophages and hallmarks of inflammatory [29]. However, PA effect on human SAT has not (to our knowledge) been investigated. Furthermore, it is known that OAT and SAT are different [30] with respect to inflammation. To this end, these studies showed that OAT releases more pro-inflammatory and less anti-inflammatory components than SAT [30, 31]. These all promoted us to investigate the effect of PA on inflammation in human SAT, lipogenesis and glucose uptake.

Accumulating evidence shows that AT is considered as one of the major sites of low grade inflammation in obese subjects [23–25], which contributes to obesity associated energy metabolic disorders such as type 2 diabetes (T2D) and cardiovascular diseases (CVDs) [4–7]. In the present study we have shown that PA inhibits the secretion of certain pro-inflammatory markers in human SAT. This is in agreement to PA effect on OAT [26], but to a lesser extent. Notably, this is in accordance with our earlier finding that PA inhibits resistin (a pro-inflammatory parameter) protein release and mRNA expression in both OAT and SAT [27]. Although It is well suggested that obesity is associated with increased infiltration of macrophages into human AT [32], which are identified via specific ATM markers, AT also contains itself macrophages. In the current study, it has been shown that PA has a great impact on ATM markers supporting the fact that PA has anti-inflammatory properties.

Most of the examined pro-inflammatory markers were not detected in the secretome of adipocytes, while the detected ones were not decreased in response to PA treatment. This may be due to a very low expression level of PA receptors GPCR-41 and -43 in adipocytes as compared to macrophages and SAT. Taking these all together, it implies that non-adipocyte cells, most likely macrophages, respond to the anti-inflammatory effect of PA.

The anti-inflammatory properties of PA are associated with other major metabolic pathways in AT, namely lipogenesis and glucose metabolism. We have found that both LPL and GLUT4 expression was up-regulated by PA. Expression of LPL and GLUT4 is known to be regulated by SREBP1c [33, 34]. Indeed, we have found an increased SREBP1c expression upon PA stimulation, suggesting that SREBP1c is responsible for the increased expression of LPL and GLUT4. These data also suggest that PA has not only an anti-inflammatory effect but also PA could have an anabolic effect similar to insulin, inducing two important metabolic pathways that are also stimulated by insulin. This is in accordance with the response of OAT to PA in our previous studies [16, 26, 35] and to the other studies where SCFAs have been found to increase insulin sensitivity [35].

The expression of the two receptors, GPRC41 and GPRC43, of PA in human AT (36), suggesting that the effects of PA on the AT might be mediated by these receptors. It has been shown that there is a unique Gi/o coupling for GPCR41, but a dual coupling exists through Gi/o and Gq proteins for GPCR43, [36]. In this study we have found that TNF-α response to PA is via Gi/o protein, suggesting that anti-inflammatory effects of PA mediated by G protein coupled receptors, while IP-10, CD163 and MMP-9 response was not mediated via Gi/o pathway. These results are in line with our earlier study [26], suggesting that both SAT and OAT employ a similar pathway (i.e. Gi/o protein coupled receptor pathway). However, our findings do not exclude the role of other pathways, such as Gq proteins and PPARγ. Therefore, further investigations are needed to dissect the underlying molecular pathway(s).

Notably, PA exhibits anti-inflammatory effects on human SAT, which is accompanied by improved expression of parameters associated with lipogenesis and glucose uptake. We also demonstrate that anti-inflammatory effects are parttially mediated by Gi/o proteins and most probably via macrophages. This finding is similar to our results what we have observed in OAT in our previous study [26, 37, 38]. The present study provides a new paradigm to understand the relationship between the microbiota and AT’s physiology and its potential power in preventing obesity-related inflammation and energy metabolic disorders. The PA anti-inflammatory properties on macrophages, the role of GPCR41 and GPCR43 receptors and other potential underlying mechanisms such as PPARγ remains to be elucidated.

These studies help us to understand better pathways involved in inflammation in ATs and macrophages for drug research and development.
